# CCS Activities Being Performed by the U.S. DOE

**DOI:** 10.3390/ijerph8020300

**Published:** 2011-01-26

**Authors:** Brian Dressel, Dawn Deel, Traci Rodosta, Sean Plasynski, John Litynski, Larry Myer

**Affiliations:** 1 National Energy Technology Laboratory, P.O. Box 10940, Pittsburgh, PA 15236, USA; E-Mails: Dawn.Deel@NETL.DOE.GOV (D.D.); Traci.Rodosta@NETL.DOE.GOV (T.R.); Sean.Plasynski@NETL.DOE.GOV (S.P.); John.Litynski@NETL.DOE.GOV (J.L.); 2 Leonardo Technologies, Inc, 320 West I Street, Benicia, CA 94510, USA; E-Mail: lrmyer@lti-global.com

**Keywords:** NETL, U.S. DOE, sequestration, geologic storage, NATCARB, depositional environments, site screening, site characterization, best practices

## Abstract

The United States Department of Energy (DOE) is the lead federal agency for the development and deployment of carbon sequestration technologies. Its mission includes promoting scientific and technological innovations and transfer of knowledge for safe and permanent storage of CO_2_ in the subsurface. To accomplish its mission, DOE is characterizing and classifying potential geologic storage reservoirs in basins throughout the U.S. and Canada, and developing best practices for project developers, to help ensure the safety of future geologic storage projects. DOE’s Carbon Sequestration Program, Regional Carbon Sequestration Partnership (RCSP) Initiative, administered by the National Energy Technology Laboratory (NETL), is identifying, characterizing, and testing potential injection formations. The RCSP Initiative consists of collaborations among government, industry, universities, and international organizations. Through this collaborative effort, a series of integrated knowledge-based tools have been developed to help potential sequestration project developers. They are the *Carbon Sequestration Atlas of the United States and Canada*, *National Carbon Sequestration Database and Geographic System (NATCARB)*, and best practice manuals for CCS including *Depositional Reservoir Classification for CO_2_; Public Outreach and Education for Carbon Storage Projects; Monitoring, Verification, and Accounting of CO_2_* *Stored in Deep Geologic Formation; Site Screening, Site Selection, and Initial Characterization of CO_2_* *Storage in Deep Geologic Formations*. DOE’s future research will help with refinement of these tools and additional best practice manuals (BPM) which focus on other technical aspects of project development.

## 1. Introduction

Our modern economy and our associated quality of life—lighting, transportation, communications, heat and air conditioning—rely fundamentally on the consumption of energy, of which approximately 85%, worldwide, comes from the combustion of fossil fuels. One of the byproducts of combustible fuels is carbon dioxide (CO_2_). Anthropogenic CO_2_ emissions and resulting increases in CO_2_ atmospheric concentrations have been generally increasing since the start of the industrial age [1]. Currently, approximately 31 billion metric tons of CO_2_ are being emitted into the atmosphere annually [2].The concentration of CO_2_ in the atmosphere is expected to increase at a rate of 1.3% annually between 2007 and 2035 [2].

While the specific links between increasing atmospheric CO_2_ concentrations and many physical and biological processes remain uncertain, there has been increasing focus over the past decade, by scientists and policymakers alike, on approaches for reducing CO_2_ emissions. One approach is to capture the CO_2_ from industrial facilities which are large emission sources. Fossil fueled power plants are prime candidates, but others include refineries, cement plants, gas processing facilities, and other industrial sources. After capture, the CO_2_ would be compressed, transported, most likely by pipeline, and injected deep in the subsurface into rock formations with the capability of storing the CO_2_ for thousands of years. This emissions reduction approach is called carbon capture and storage (CCS). DOE’s Office of Fossil Energy manages a Carbon Sequestration Program through NETL which focuses on research and development of CCS technologies to reduce CO_2_ emissions.

DOE’s Carbon Sequestration Program is focused on geologic storage of captured CO_2_ that would otherwise be emitted to the atmosphere. Applied research is being conducted to develop and test different approaches to CCS. Technology testing and development through the Regional Carbon Sequestration Partnership (RCSP) Initiative and other entities will be instrumental to the commercial deployment of CCS. DOE’s vision is to fully understand the available CCS options, cost factors, environmental implications, and technological options.

## 2. DOE’s Carbon Sequestration Program

**Figure 1.**
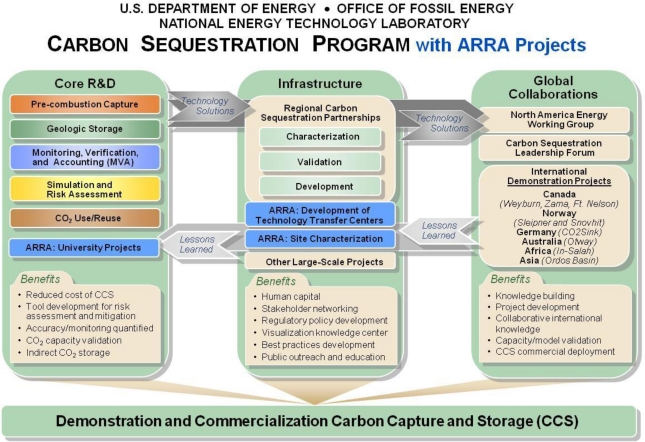
DOE’s Carbon Sequestration Program.

In 1997, DOE established the Carbon Sequestration Program (Program), which is administered by the Office of Fossil Energy and implemented through NETL to move CCS technologies toward commercialization. The Program encompasses all aspects of CCS and has engaged government and private sector partners that have expertise in CCS technology. The Program covers three key elements for technology development (Figure 1): core research and development (R&D), infrastructure, and global collaborations. The R&D element is driven by industry’s technology needs and categorizes those needs into five focal areas to more efficiently obtain solutions that can then be tested and deployed in the field. The infrastructure element includes the RCSPs and other small and large-volume field tests in different geologic formation classes where validation of various CCS technology options and their efficacy are being confirmed. The global collaborations element benefits from technology solutions developed in the R&D and infrastructure elements and, in turn, feeds lessons learned into infrastructure and R&D. Lessons learned from the infrastructure element are also fed back into R&D to guide future applied research and development of CCS technologies. Funds from the American Recovery and Reinvestment Act (ARRA) of 2009 were recently utilized by the program to develop CCS technology training centers, conduct additional site characterization studies and to fund small research projects related to CCS.

The Program strives to develop fossil fuel conversion systems that offer 90% CO_2_ capture with a less than 10% increase in the cost of energy services for pre-combustion carbon capture ready for wide scale deployment in the 2020 timeframe. In addition, the Program aims to achieve 99% storage permanence while validating storage potential within +/− 30%. Attaining these goals will require an integrated approach to address CCS challenges through R&D projects, participation in working groups, and the RCSP Initiative. Development of these technologies will help address future challenges to overcome a multitude of economic, social, and technical challenges, including cost-effective CO_2_ capture through successful integration with fossil fuel conversion systems; effective CO_2_ monitoring and verification; permanence of underground CO_2_ storage; and public acceptance.

The commercial deployment of CCS faces many challenges. Technical challenges include the development of lower cost capture technologies, accurate estimates of geologic storage potential, and evaluating the permanence of injected CO_2_. Legal and social issues include developing a regulatory framework with regards to the permitting and treatment of CO_2_ once it is injected into a geologic reservoir, developing infrastructure such as pipelines, developing a workforce trained in CCS, and the legal framework for the liability and ownership of the pore space and injected CO_2_. Additionally, commercial deployment of CCS will require public education on the benefits of CCS. The Program is developing best practice manuals from lessons learned to help transfer knowledge gained to the private sector.

## 3. Regional Carbon Sequestration Partnerships

The RCSPs are public/private cooperative efforts tasked with developing guidelines and testing the most suitable technologies, regulations, and infrastructure needs for CCS in the United States and Canada. The seven RCSPs that form this initiative currently include more than 400 state agencies, universities, and private companies, spanning 43 states, and four Canadian provinces.

The RCSPs’ initiative consists of three distinct phases of work: (1) Characterization Phase (2003–2005); (2) Validation Phase (2005–2011); and (3) Development Phase (2008–2018+). The Characterization Phase began in September 2003 with the seven RCSPs working to develop the necessary framework to validate and potentially deploy CCS technologies. At the end of the Characterization Phase, the RCSPs had succeeded in establishing a national network of companies and professionals working to support CCS deployments, creating a National Carbon Sequestration Database and Geographic Information System (NATCARB), and raising awareness and support for CCS as a green house gas (GHG) mitigation option. Fact sheets for some of the projects conducted by the RCSPs and NETL are available at http://www.netl.doe.gov/technologies/carbon_seq/refshelf/refshelf.html.

The Validation Phase focuses on validating the most promising regional opportunities to deploy CCS technologies by building upon the accomplishments of the Characterization Phase. Efforts are being conducted to (1) validate and refine current reservoir simulations for CO_2_ storage projects; (2) collect physical data to confirm CO_2_ storage potential and injectivity estimates; (3) demonstrate the effectiveness of monitoring, verification, and accounting (MVA) technologies; (4) develop guidelines for well completion, operations, and abandonment; and (5) develop strategies to optimize the CO_2_ storage potential of various geologic formations. The Validation Phase consists of 20 geologic injection tests.

The Development Phase builds on the information generated in the Characterization and Validation Phases and involves the injection of 1 million tons or more of CO_2_ by each RCSP into regionally significant geologic formations. These large-volume injection tests are designed to demonstrate that CO_2_ storage sites have the potential to store regional volumes of CO_2_ emissions safely, permanently, and economically for hundreds of years. Development Phase tests will result in a better understanding of commercial scale CCS projects and of regulatory, liability, and ownerships issues associated with these projects. These projects will provide a firm foundation for commercialization of large-scale CCS.

## 4. Development of the Carbon Sequestration Atlas of the United States and Canada and the NATCARB Database

DOE manages the development of a powerful, user-friendly database that supplies regions of the country with valuable information regarding CCS. The database, called the National Carbon Sequestration Database and Geographic Information System (NATCARB), was originally designed to assess the potential for geologic CO_2_ storage in five Midwestern states (Indiana, Illinois, Kansas, Kentucky, and Ohio). With the implementation of the RCSP Initiative, the database was expanded to cover the entire U.S. and parts of Canada by linking the seven RCSPs and various public databases. NATCARB provides web-based data access to CO_2_ stationary sources, potential geologic CO_2_ storage data, infrastructure information, supporting analytical tools for CO_2_ storage resource estimation, and CCS cost estimation. NATCARB addresses the broad needs of all users, and includes not only geographical information systems (GIS) and database query tools for the high-end technical user, but also simplified displays for the general public, employing readily available web tools such as Google Earth™ and Google Maps™. Data are generated, maintained, and enhanced locally at the RCSP level, or at specialized data warehouses and public servers. They are assembled, accessed, and analyzed in real-time through a single geoportal. NATCARB is available through the NETL/DOE website at http://www.netl.doe.gov/technologies/carbon_seq/natcarb/index.html.

## 5. Development of a Depositional Classification Scheme for CO_2_ Reservoirs

Through NETL, DOE has recently released a comprehensive manual, titled *Geologic Storage Formation Classification: Understanding Its Importance and Impact on CCS Opportunities in the United States*, to better understand the characteristics of potential geologic storage formations as a component of CCS. This desk reference is intended to:

- Assist with an understanding of basic geological principles and terminology associated with potential CO_2_ geologic storage in formations.
- Show the importance of geologic depositional systems in determining the internal architecture of such formations, thus making it possible to predict the general behavior of the injected CO_2_.
- Establish the importance of using the geologic depositional system to assess existing and future research, design, and demonstration needs related to storing CO_2_ in different depositional environments.
- Focus the efforts of DOE on potential reservoirs in depositional environments that have not been previously investigated.

Three major rock types might be targeted by future developers of CCS projects for storage formations: igneous, metamorphic, and sedimentary. Each major type of rock was formed under different conditions, and their potential for CO_2_ storage varies based on the necessary criteria of:

- **Capacity**, based on the porosity or openings within a rock, often called “pore space”.
- **Injectivity**, dependent on the permeability or the relative ease with which a fluid or gas can move within the pore space(s) of a rock.
- **Integrity**, the ability to confine a fluid or gas within a geologic unit, is of primary importance, because without impermeable seals, fluids will take the path of least resistance and move to a lower pressure area, including the surface.

The answers to questions concerning capacity, injectivity, and integrity can be learned, in part, by reservoir characterization of the formations in the area of the proposed geologic storage site. Reservoir characterization is an evolving science that integrates many different scientific disciplines (geology, geophysics, mathematical modeling, computational science, seismic interpretation, well log, and core analysis, *etc.*) in order to build a conceptual model of a formation. The decision to select a particular geologic unit for geologic storage usually depends on a detailed understanding of the reservoir characteristics and the behavior and fate of the injected fluids and their impact on the geologic strata receiving the fluids. Critical factors include economic analysis of the location of the site, distance from the CO_2_ source to the site, depth of the reservoir (which influences drilling and injectivity of CO_2_), the volume of CO_2_ that the site can contain, the trapping mechanism and sealing capacity, and the ultimate fate of the stored CO_2_. Many of these issues will be affected by the different classes of reservoirs being targeted for injection.

Most CO_2_ geologic storage targets are sedimentary rocks (clastics and carbonates), where CO_2_ storage is in the pore space between grains, which are most often filled with undrinkable saline water. Igneous formations, which cover more of the Earth’s surface than sedimentary formations, offer potentially great geologic storage sites because of their total volume both on continents and under the oceans, but are mostly untested. Coal seams are considered both sedimentary and metamorphic and have their own unique properties. The most important storage mechanism for coal is its preferential ability to absorb CO_2_ directly on its surface. This situation differs from other sedimentary and igneous formations where the CO_2_ occupies the pore space.

One major goal of the Program is to classify the depositional environments of various formations that are known to have excellent reservoir properties and are amenable to geologic CO_2_ storage. This is being accomplished through the implementation of 28 CO_2_ injection field projects in collaboration with the RCSP Initiative and ten American Recovery and Reinvestment Act of 2009 (Recovery Act) projects focused on the characterization of geologic formations as sites for possible commercial CCS development. DOE proposes a geologic depositional classification system for CO_2_ storage to better understand how the field work being conducted fulfills the need to test these different classes of depositional systems and determine what future R&D projects are still needed.

While geologic formations are infinitely variable in detail, they have been classified by geologists and engineers in the petroleum industry by their trapping mechanism, the hydrodynamic conditions (mechanical forces that produce), lithology (physical characteristics), and more recently by their depositional environment (how they were formed). The depositional environment influences how formation fluids are held in place, how they move, and how they interact with other formation fluids and solids (minerals). For the purposes of geologic storage, the geologic formation/reservoir classification system has been expanded to include unconventional reservoirs, such as coal seams, and igneous formations, like stacked basalts. The reservoir classification scheme developed for CO_2_ storage, based on depositional environments, is presented as Table 1.

For fluid flow in porous media, knowledge of how depositional systems formed and directional tendencies imposed by the depositional environment can influence how fluids flow within these systems and how CO_2_ in geologic storage would be anticipated to flow in the future. Although diagenesis has modified fluid flow paths in the intervening millions of years, the basic architectural framework created during deposition remains; geologic processes that exist today also existed when the sediments were initially deposited. Analysis of modern day depositional analogs and evaluation of core, outcrops, and well logs from ancient subsurface formations provide an indication of how formations were deposited and how fluid flow within the formation is anticipated to flow.

The DOE is gathering data and developing a database of regional reservoirs and associated properties for each type of depositional environment. This data could be utilized by site developers and property owners to develop risk assessments and business models for CCS and to better define costs for geologic storage and determine the type and quality of geologic reservoirs in a region. DOE’s goal is to characterize the different depositional environments with drilling, subsurface geophysics, chemical analysis, geomechanical analysis of the rocks, and conducting both small- and large-scale CO_2_ injection tests.

The results of DOE’s initial evaluation indicate that reservoir characterization (with the ability to store >30 million tons of CO_2_) has not been completed for shelf clastic, reef, and coal environments. Small-scale injection tests have not been performed on fluvial deltaic, eolian, and turbidite sedimentary environments. Large-scale injection tests have not been performed on deltaic, strandplain, shelf carbonate, eolian, turbidite, basalt large igneous providences (LIP), and coal. Three highly experimental reservoirs (fractured shales, basalts mid-oceanic ridge [MOR], and offshore turbidites) have not been evaluated. The evaluated projects are in various states of completion—some investigations are completed and some just started. Understanding the impacts of different reservoir classes on CO_2_ storage supports DOE’s efforts to develop the knowledge and tools necessary for commercialization of CCS technologies throughout the United States. Using lessons learned from the behavior of CO_2_ in reservoirs from these geologic investigations and their known depositional environments is important in developing an understanding for similar depositional environments being considered for storage (Table 2).

## 6. Technology Transfer and the Development of Best Practice Manuals

Through the various projects in the Program, lessons learned have been documented in a series of best practice manuals (BPMs) that serve as the basis for the design and implementation of commercial CCS projects. As of August 2010, DOE has released three BPMs: (1) “Public Outreach and Education for Carbon Storage Projects,” (2) “Site Screening, Selection, and Characterization for Storage of CO_2_ in Deep Geologic Formations,” and (3) “Monitoring, Verification, and Accounting of CO_2_ Stored in Deep Geologic Formations.”

### Public Outreach and Education for Carbon Storage Projects

The objective of the Public Outreach and Education for Carbon Storage Projects Best Practices Manual is to communicate lessons learned and to recommend best practices emerging from the public outreach conducted by the seven RCSPs. The manual is intended to assist project developers in understanding and adopting best practices in outreach to support CO_2_ storage projects. Although project developers are the primary audience for this document, other stakeholders may find the contents useful. Early CO_2_ storage projects have been highly visible, and their success will likely impact future CO_2_ storage projects.

The primary lesson learned from the RCSPs’ experience is that public outreach should be an integrated component of project management. Conducting effective public outreach will not necessarily ensure project success, but underestimating its importance can contribute to delays, increased costs, and community ill will. Effective public outreach involves listening, sharing information, and addressing concerns through proactive community engagement. Public outreach begins at the onset of the project, continues through the close of the project, and involves each individual on the project team.

The RCSPs’ concept of public outreach involves efforts to understand, anticipate, and address public perceptions and concerns about CO_2_ storage in a community being considered for a project. Ideally, public outreach can lead to a mutually beneficial outcome where project developers move ahead with the support of well-informed stakeholders who are comfortable with the project benefits and potential risks and trust the project team. As described in this BPM and shown in Table 3, the RCSPs have proposed the 10 best practices for CCS public outreach.

## 7. Site Screening, Site Selection, and Initial Characterization for Storage of CO_2_ in Deep Geologic Formations

**Figure 2.**
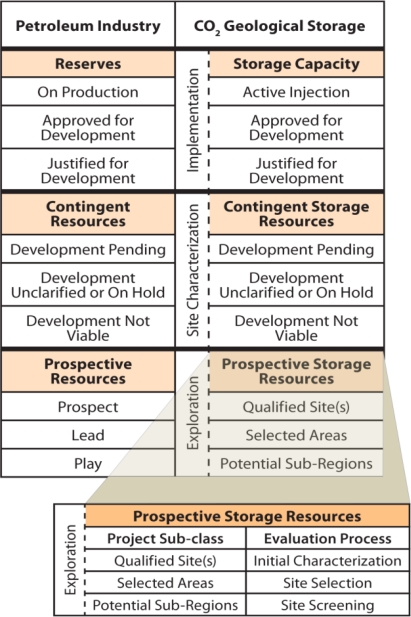
Comparison of Petroleum Industry Classification and Proposed CO_2_ Geologic Storage Classification. Adapted from SPE/WPC/AAPG/SPEE Resource Classification System. (© 2007 Society of Petroleum Engineers, Petroleum Resource Management System).

Another in the series of BPMs developed is the “Site Screening, Site Selection, and Initial Characterization for Storage of CO_2_ in Deep Geologic Formations,” which includes a series of process diagrams and guidelines for site screening, site selection, and initial characterization. This document is based on the lessons learned from the RCSPs through the Validation Phase and integrates the analyses into a proposed geologic storage framework. The proposed classification framework is divided into three phases: Exploration Phase, Site Characterization Phase, and Implementation Phase. The Exploration Phase classifies storage estimates for prospective storage and classifies the site based on the level of analyses conducted. The Exploration Phase has three project sub-classes: Potential Sub-Regions, Selected Areas, and Qualified Site(s). These sub-classes correspond to three stages of evaluation during the Exploration Phase: site screening, site selection, and initial characterization (Figure 2). The most important objectives of the Exploration Phase are to lay the groundwork to ensure safe storage of CO_2_ and compliance with the Underground Injection Control (UIC) program requirements.

**Figure 3.**
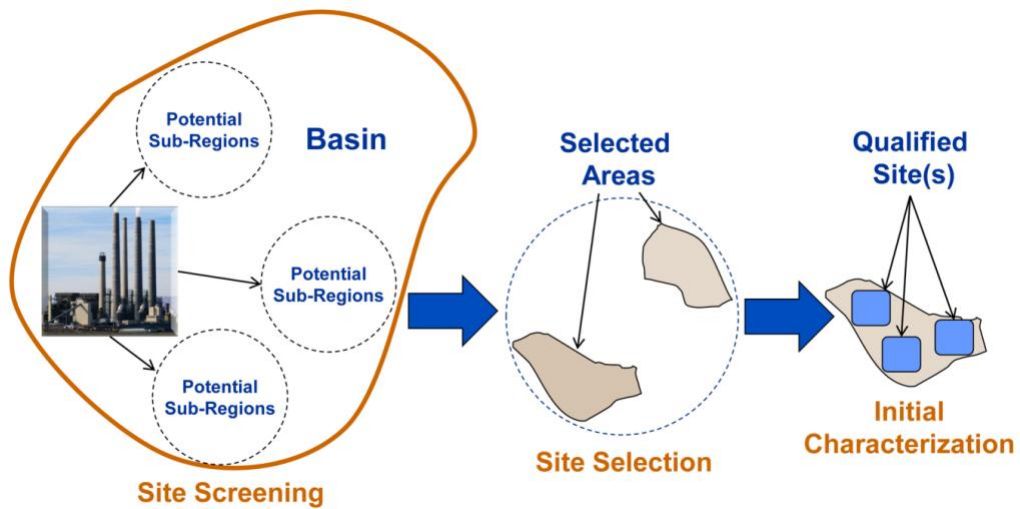
CCS screening process.

The primary goal of the Exploration Phase is to pare down a large region into a select few sites as seen in Figure 3 below. The ultimate goal of this process is to identify sites with highest potential for storage, and help eliminate from consideration those that are less preferable. The site screening stage evaluates existing data and resources from sub-regional data thorough a series of analyses on critical components to assess storage potential within a potential sub-region. These results in a set of selected areas that are then ranked based on criteria established during project definition, and the highest ranking selected areas advance to the site selection stage. The selected areas are then further analyzed through the site selection components, and the most promising qualified sites proceed to the final stage in the Exploration Phase—initial characterization.

**Figure 4.**
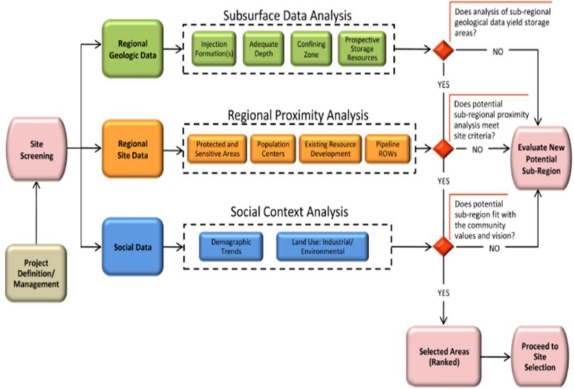
Process flow chart for site screening and initial characterization.

The Site Screening process diagram in Figure 4 describes analyses that should be conducted on elements within each of the three components—regional geologic data, regional site data, and social data. Once the analysis is completed for each of the three components, a decision gate is reached. A “yes” response to all three analyses advances a selected area within the potential sub-region to the next stage and a “no” response at any decision gate will result in a new potential sub-region being selected with the process beginning over again. The selected area will then proceed through the next series of analyses in the site selection evaluation stage.

In order to assist future project developers, a set of guidelines (Table 4) has been developed for each of the elements within the component being analyzed for all three stages of evaluation. The guidelines presented in the manual are not intended to be prescriptive but provide future project developers with an understanding of the level of work necessary to further mature a project. Table 4 includes the guidelines for site screening.

Upon completion of the site screening process, a selected area will be further evaluated during the site selection process. During site selection, five components will be analyzed. Existing data and analyses from site screening will be augmented with proprietary or other purchased data to evaluate both technical and nontechnical components, subsurface geologic data, regulatory requirements, model data, site data, and social data. Completion of each analysis will lead to a decision gate. A “yes” response will result in a list of qualified sites that will then be evaluated based on an economic feasibility plan. A “no” response at any decision gate will result in an alternative selected area being selected, and the analysis will begin again. Once it successfully proceeds through the decision gates, the selected area will be included on a list of qualified sites to be prioritized based on criteria developed during project definition, and the highest priority qualified site will proceed to the next evaluation stage—initial characterization. Process maps and site selection guidelines can be found on the NETL/DOE website: http://www.netl.doe.gov/technologies/carbon_seq/refshelf/BPM-SiteScreening.pdf.

The final stage of evaluation in the Exploration Phase is initial characterization. The distinction between the initial characterization stage within the Exploration Phase and within the Site Characterization Phase is based on the level of additional funding and detailed analyses needed for the site to prepare an evaluation for potential implementation. During initial characterization, sites identified in site selection will be evaluated using five technical and nontechnical elements, including (1) baseline data, (2) regulatory requirements, (3) model data, (4) social data, and (5) site development plan. The site would be evaluated according to the evaluation criteria and a determination would be made to either collect additional data and elevate the site to the Site Characterization Phase or leave it ranked as a qualified site. If the qualified site advances to the Site Characterization Phase, then the storage estimates would be considered contingent storage resource. Initial characterization process maps and guidelines can be found on the NETL/DOE website.

The site screening, site selection, and initial characterization manual will be periodically updated based on new lessons learned from the Development Phase of DOE projects. The geologic storage classification framework will also be updated as definitions and project status guidelines are further developed and refined for the Site Characterization and Implementation Phases.

## 8. Monitoring, Verification, and Accounting of CO_2_ Stored in Deep Geologic Formations

Reliable and cost-effective MVA techniques are an important part of making CO_2_ geologic storage a safe, effective, and acceptable method for GHG control. MVA of geologic storage sites is expected to serve several purposes, including addressing safety and environmental concerns; inventory verification; project and national accounting of GHG emissions reductions at geologic storage sites; and evaluating potential regional, national, and international GHG reduction goals.

Each geologic storage site varies significantly in risk profile and overall site geology, including target formation depth, formation porosity, permeability, temperature, pressure, and seal formation. MVA packages selected for commercial-scale projects should be tailored to site-specific characteristics and geologic features. In general, the goals of an MVA best practice manual for geologic storage are to:

- Improve understanding of storage processes and confirm their effectiveness.
- Evaluate the interactions of CO_2_ with formation of solids and fluids.
- Assess environmental, safety, and health impacts in the event of a leak to the atmosphere.
- Evaluate and monitor any required remediation efforts should a leak occur.
- Provide a technical basis to assist in legal disputes resulting from any impact of sequestration technology (groundwater impacts, seismic events, crop losses, *etc.*).

The life cycle of a geologic storage project involves four phases. Monitoring activities will vary among these phases:

- *Pre-Operation Phase*: Project design is carried out, baseline conditions are established, geology is characterized, and risks are identified.
- *Operation Phase*: Period of time during which CO_2_ is injected into the storage reservoir.
- *Closure Phase*: Period after injection has stopped (wells are abandoned and plugged, equipment and facilities are removed, and previously determined site restoration is accomplished). Only necessary monitoring equipment is retained.
- *Post-Closure Phase*: Period when ongoing monitoring is used to demonstrate that the storage project is performing as expected until it is safe to discontinue further monitoring. Once it is satisfactorily demonstrated that the site is stable, monitoring will no longer be required except in the unlikely event of release, regulatory requirements, or other matters that may require new information about the status of the storage project.

This BPM evaluated the different available technologies for use in MVA. The technologies were evaluated for their effectiveness of monitoring atmospheric concentrations of CO_2_, near surface CO_2_, and subsurface CO_2_. The evaluated technologies were subdivided as primary technologies, secondary technologies, and potential additional MVA technologies.

- Primary technologies are considered proven technologies capable of satisfying the monitoring requirements under the United States Environmental Protection Agencies underground injection control (UIC) regulations for Class I (non-hazardous), Class II(enhanced oil recovery operations), and Class V (experimental) injection wells and meet a goal of 99 percent containment by 2015. These technologies have been utilized in the petroleum industry and for geologic characterization.
- Secondary technologies are technologies that show promise but would need to demonstrate that they are sufficiently precise and quantitative to detect, locate, and quantify emissions as part of a CCS monitoring program.
- Potential additional MVA technologies are promising additional technologies being developed to better understand the long-term behavior of CO_2_ in a broad portfolio of potential reservoirs types. This also includes improvements of existing technologies to allow for detailed monitoring of CO_2_ in GS.

In order to implement effective controls on injection well completion, injection rates, and well head and formation pressures, specific monitoring objectives were recommended by Benson *et al.* (2004) [3], including:

- Establishing baseline conditions from which the impacts of CO_2_ storage can be assessed.
- Assessing the integrity of shut-in, plugged, or abandoned wells.
- Monitoring to ensure injection effectiveness.
- Monitoring to detect the location of the injected CO_2_ plume.
- Comparing model predictions to monitoring data.
- Detecting and quantifying leakage from the storage formation to other strata or the surface.
- Assessing health, safety, and environmental impacts of leakage.
- Monitoring to detect micro-seismicity associated with CO_2_ injection.
- Monitoring to aid in the design and evaluation of remediation efforts, if needed.
- Evaluating interactions with, or impacts on, other geologic resources.
- Reassuring the public, where visibility and transparency are of prime importance_3_.

The monitoring requirements may change through the different phases of the project. This is dependent on the project’s needs and site-specific conditions. The recommended steps in the BPM for selection of suitable geophysical techniques include:

- Developing geologic models for the sequestration site that include the reservoir, the seals, and overlying geology, aquifer(s), vadose zone, and surface.
- Performing reservoir simulations of the sequestration processes of interest, such as prediction of changes and the distribution of fluid phases resulting from CO_2_ injection.
- Using the geologic model and results of reservoir simulations to perform numerical simulations to predict the response of candidate geophysical and geochemical monitoring techniques.

The goal of this BPM is to provide information to limit unnecessary burden on owners, operators, or permitting agencies and provide a strong foundation for national consistency in permitting and safe operation of geologic storage projects.

Recent regulatory developments through the U.S. EPA have focused on finalizing rules for a new Class of UIC injection well for CO_2_ storage projects and monitoring requirements under the Clean Air Act. DOE plans to update the MVA BPM to account for additional monitoring requirements once these rules become final (http://water.epa.gov/type/groundwater/uic/wells_sequestration.cfm).

## 9. Conclusions

Through NETL’s RCSP Initiative, informational tools are being developed to promote successful deployment of CCS technology as a GHG mitigation option. As part of this effort, the RCSPs provide information from their regional characterization efforts and field projects to support the development of NATCARB and the BPMs. NATCARB is updated in real-time as the RCSPs perform and obtain results from their field tests allow real time access to the most recent data that the RCSPs have available. The *Carbon Sequestration Atlas of the U.S. and Canada* is updated and published every two years. The BPMs will be updated as results from the RCSP field tests are analyzed and published. It is also anticipated that these BPMs will be updated as the technology and information matures.

This document was developed from several different studies/documents that were sponsored by NETL and the RCSP Initiative. A list of the documents, in addition to the cited references, is included in Appendix.

## Figures and Tables

**Table 1.** Proposed depositional environments classification scheme.

**Reservoir Depositional Classification Schematic**						
Rock Classification Lithology	Geoscience Institute for Oil and Gas Recovery Research Classification in 1991			DOE’s Oil Reservoir Classification from 1990’s	Sequestration Formation Classification 2010	
					Storage	Seals
Sedimentary	Clastic Reservoirs	Delta	Delta/Fluvial-Dominated	Class I Reservoirs	Deltaic	Shales (fine terrigenous materials—clays as well as from carbonates) Deposited in Lacustrine, Fluvial, Alluvial, Near Shore and Open Ocean Marine Environments
			Delta/Wave-Dominated			
			Delta/Tide-Dominated		Coal/Shale	
			Delta/Undifferentiated			
		Fluvial	Fluvial/Braided Stream	Class 5 Reservoirs	Fluvial	
			Fluvial/Meandering Stream			
			Fluvial/Undifferentiated			
		Alluvial Fan			Alluvial	
		Strandplain	Strandplain/Barrier Cores and Shorefaces	Class 4 Reservoirs	Strandplain	
			Strandplain/Back Barriers			
			Strandplain/Undifferentiated			
		Turbidites	Slope-Basin	Class 3 Reservoirs	Turbidite	
			Basin			
		Eolian — Wind Blown: Clastics and/or Carbonates			Eolian	Evaporites (from various Lithology Deposited in Arid Settings)
		Lacustrine — Lake Deposited: Clastics, Carbonates, Evaporites			Lacustrine	
		Shelf			Shelf	
	Carbonate Reservoirs	Peritidal	Dolomitization			
			Massive Dissolution			
			Other			
	Carbonate (>50% Carbonate content but can contain Terrigenous materials — sand, feldspar, non-carbonate boulders and evaporites)	Shallow Shelf/Open	Dolomitization	Class 2 Reservoirs	Shallow Shelf	
			Massive Dissolution			
			Other			
		Shallow Shelf/Restricted	Dolomitization			
			Massive Dissolution			
			Other			
		Reef	Dolomitization		Reef	
			Massive Dissolution			
			Other			
		Shelf Margin	Dolomitization			
			Massive Dissolution			
			Other			
		Slope-Basin	Other			
Igneous	Basalts				Basaltic	
					Interflow Zones	
	Granitic					
Metamorphic						

**Table 2.** Matrix of NETL CO2 geologic storage projects and geologic formation classes.

**Geologic Formation Classes**	High Potential					Medium Potential				Lower or Unknown Potential	
	Deltaic	Shelf Clastic	Shelf Carbonate	Strandplain	Reef	Fluvial Deltaic	Eolian	Fluvial & Aluvial	Turbidite	Coal	Basalt
											(LIP)
*Large Scale*	–	1	–	–	1	3	–	1	–	–	–
*Small Scale*	3	2	4	1	2	–	–	2	–	5	1
*Characterization*	1	–	8	6	–	3	3	2	2	–	1

Notes: The number in the cell is the number of investigations per depositional environment.

Large Scale Field Tests—Injection of over 1,000,000 tons of CO2.

Small Scale Field Tests—Injection of less than 500,000 tons of CO2.

Site Characterization—Characterize the subsurface at a location with the potential to inject at least 30,000,000 tons of CO_2_.

Reservoir potentials were inferred from petroleum industry data and field data from the sequestration program.

**Table 3.** Ten best practices for public outreach.

**Best Practice**	**Description**
Integrate Outreach with Project Management	By including outreach in the critical path of a CO_2_ storage project, outreach activities will be more effective, in sync with other key project stages, and beneficial to the overall project; a key component is building in the time necessary to accomplish the various steps in advance of engaging the public.
Establish a Strong Team	It is essential to establish a clearly defined structure that delineates roles and responsibilities covering both internal and external communication and includes individuals who are knowledgeable about the technical details of the project, as well as individuals who have backgrounds in communication, education, and community relations.
Identify Key Stakeholders	Early CO_2_ storage projects are being carried out in the context of national debates on climate change mitigation and, as a result, stakeholders may come from an area that extends beyond the project’s location and regulatory jurisdiction. It is critical to identify all stakeholders in the project lifecycle. At the local level, these may include elected and safety officials, regulators, landowners, citizens, civic groups, business leaders, media, and community leaders. At the national level, these may include Government agencies, Congressional leaders, committee/subcommittee chairs and key staff, environmental groups, and the financial and legal community.
Conduct Social Characterization	Social characterization is an approach for gathering and evaluating information to obtain an accurate portrait of stakeholder groups, their perceptions, and their concerns about CO_2_ storage. This approach can identify the factors that will likely influence public understanding of CO_2_ storage within a specific community. The information gathered will enable the project team to develop better insights into the breadth of diversity among community members, local concerns and potential benefits, and assist in determining which modes of outreach and communication will be most effective.
Develop a Strategy and Communication Plan	The outreach strategy and communications plan ties together the information, planning, and preparation. The outreach strategy is tailored to the stakeholder needs and concerns of a particular CO_2_ storage project. Specifics will include outreach objectives, outreach tasks, and events that coincide with the project stages, a timeline for outreach activities, and the roles and responsibilities of the outreach team. The outreach strategy will also identify key stakeholders and messages, and the timelines, roles, and responsibilities for producing outreach materials and managing outreach events. A component of the outreach strategy is a communications plan that focuses on representing the project directly to the public and through the media.
Develop Key Messages	CO_2_ storage involves advanced science related to climate change, geology, and other fields of study; public policy related to energy, environment, and the economy; and issues related to risk, safety, and financial assurance. Therefore, identifying a set of key messages that can be consistently repeated in outreach activities and materials can help stakeholders develop a clearer understanding of the project and how their concerns will be addressed.
Develop Materials Tailored to Audiences	The development of outreach materials involves consideration of the intended audience. The amount of information and level of technical detail provided must be tailored to match the audience’s degree of interest, education, and time constraints. Any concerns that have been identified, including perceived risks, should be addressed in language and formats suited to the intended audiences.
Proactively Manage the Program	Outreach programs should be actively managed to ensure that consistent messages are being communicated and that requests for information are fulfilled throughout the project lifecycle. The identification of an outreach leader or coordinator to manage, coordinate, and direct outreach is crucial for project success. The outreach lead will be supported in their efforts by the outreach team and other project team members. As a project unfolds, public perception will to be influenced by the extent to which the project and the project team are well coordinated and responsive.
Monitor the Program and Public Perceptions	Monitoring the performance of the outreach program allows the project team to stay abreast of how the community perceives the project and gauge the effectiveness of the outreach activities. Monitoring can also help identify any misconceptions about the project or CO_2_ storage and develop outreach strategies to correct them.
Refine the Program as Warranted	The outreach team must be ready to adapt to changes in information about the site, unexpected events, and other conditions that may have a strong influence on the public’s perception of CO_2_ storage during project implementation.

**Table 4.** Guidelines for site screening.

**COMPONENT**		**ELEMENT**	**GUIDELINES FOR SITE SCREENING**
**Regional Geologic Data**	**Subsurface Data Analysis**	Injection Formation(s)	Identify regional and sub-regional injection formation types. Utilize readily accessible data from public sources (e.g., state geological surveys, NATCARB, the Regional Sequestration Partnerships, published and open-file literature, academic sources) or acquired from private firms. Data gathered should include regional lithology maps, injection zone data (thickness, porosity, permeability), structural maps, information about structure closure and features that might compartmentalize the reservoir such as stratigraphic pinch outs, regional type logs, offset logs, petrophysical data, and regional seismicity maps.
		Adequate Depth	Assessment of minimum depth of the injection zone to protect USDWs is required; in addition depths greater than 800 m generally indicate CO_2_ will be in a supercritical state and may be more cost-effectively stored. Shallow depths (generally <800 m) may add to the risk profile because (1) CO_2_ could be in gas phase and (2) the injection zone may be closer to USDW.
		Confining Zone	Candidate injection zones should be overlain by a confining zone comprised of one or more thick and impermeable confining intervals of sufficient lateral extent to cover the projected aerial extent of the injected CO_2_. Confining zones can be identified on a regional basis from the same types of information used to identify injection formations. Wells that penetrate potential confining zones should be identified and included in the risk assessment; this information can be obtained from state oil and gas regulatory agencies. Faulting and folding information that may impact confining zone integrity should be mapped along with potential communication pathways. Confining zone integrity may be validated by presence of nearby hydrocarbon accumulations.
		Prospective Storage Resources	Candidate CO_2_ storage formations should contain enough Prospective Storage Resources beneath a robust confining zone for the volume of CO_2_ estimated during Project Definition and the displaced fluids. Prospective Storage Resources (and injectivity if permeability data is available) should be estimated at the sub-regional scale utilizing existing data (e.g., NATCARB, and state geological surveys) to populate basic numerical models.
**Regional Site Data**	**Regional Proximity Analysis**	Protected and Sensitive Areas	Identify environmentally sensitive areas using U.S. Environmental Protection Agency, U.S. Department of Interior, U.S. Forest Service and U.S. Bureau of Land Management GIS systems. Assess the potential for conflicts with siting of pipeline routes, field compressors and injection wells. In addition, evaluate potential for other surface sensitivities utilizing maps for other hazards (e.g., flood, landslide, tsunami).
		Population Centers	Identify population centers using state and federal census data. Assess the potential for conflicts with siting of carbon storage projects.
		Existing Resource Development	Identify existing resource development, including wells that penetrate the confining zone, using data from state and federal oil and gas, coal, mining and UIC and natural resource management offices. Assess the potential for conflicts between siting of carbon storage projects and existing or prospective mineral leases as well the availability of complementary or competing infrastructure.
		Pipeline ROWs	Identify all pipelines and gathering lines/systems. Assess potential for conflicts in routing of pipelines to carbon storage projects as well as the potential for use or access to existing pipeline right-of-ways (ROWs). Identify other ROWs (e.g., powerlines, RR's highways) and assess potential for synergies or conflicts in siting carbon storage projects. This data can be found through commercial and government sources.
**Social Data**	**Social Context Analysis**	Demographic Trends	Describe communities above and near candidate Sub-Regions by evaluating readily available demographic data and media sources. To the extent possible, assess public perceptions of carbon storage and related issues; develop an understanding of local economic and industrial trends; and begin to identify opinion leaders.
		Land Use: Industrial and Environmental History	Describe the trends in land use, industrial development and environmental impacts in communities above or near candidate Sub-Regions by evaluating sources such as online media sites, regulatory agencies, corporate websites, local environmental group websites, and other sources. Begin to assess community sensitivities to land use and the environment.
**Complete Site Screening**		Selected Area	Develop a list of potential Selected Areas and rank based on criteria established in Project Definition.

## Appendix

### Source Documents

1. *Carbon Sequestration Technology Roadmap and Program Plan 2005*; United States Department of Energy, National Energy Technology Laboratory: Pittsburgh, PA, USA, May 2005.
2. *Carbon Sequestration Program Overview*; United States Department of Energy, National Energy Technology Laboratory: Pittsburgh, PA, USA, 2010. Available online: http://www.netl.doe.gov/technologies/carbon_seq/overview/index.html (accessed on 2 November 2010).
3. *Carbon Sequestration Program Goals*; United States Department of Energy, National Energy Technology Laboratory: Pittsburgh, PA, USA, 2010. Available online: http://www.netl.doe.gov/technologies/carbon_seq/overview/program_goals.html (accessed on 2 November 2010).
4. *Carbon Sequestration Atlas of the United States and Canada*; United States Department of Energy, National Energy Technology Laboratory: Pittsburgh, PA, USA, November 2010.
5. *Geologic Storage Formation Classification: Understanding Its Importance and Impacts on CCS Opportunities in the United States*; United States Department of Energy, National Energy Technology Laboratory: Pittsburgh, PA, USA, September 2010.
6. *Best Practices Manual for: Public Outreach and Education for Carbon Storage Projects*; United States Department of Energy, National Energy Technology Laboratory: Pittsburgh, PA, USA, December 2009.
7. *Draft Best Practices Manual for: Site Screening, Site Selection, and Initial Characterization for Storage of CO_2_ in Deep Geologic Formations*; United States Department of Energy, National Energy Technology Laboratory: Pittsburgh, PA, USA, June 2010.
8. *Best Practices Manual for: Monitoring, Verification, and Accounting of CO_2_ Stored in Deep Geologic Formations*; United States Department of Energy, National Energy Technology Laboratory: Pittsburgh, PA, USA, January 2009.
